# Supplementary material for: Combinatorial Glycomic Analyses to Direct CAZyme Discovery for the Tailored Degradation of Canola Meal Non-Starch Dietary Polysaccharides
Source: Microorganisms. 2020 Nov 29;8(12):1888. doi: 10.3390/microorganisms8121888 (PMC7761036; doi:10.3390/microorganisms8121888)
Supplement: Supplementary file 1 [file microorganisms-08-01888-s001.zip › FigS2_fractionation_yield.pdf]

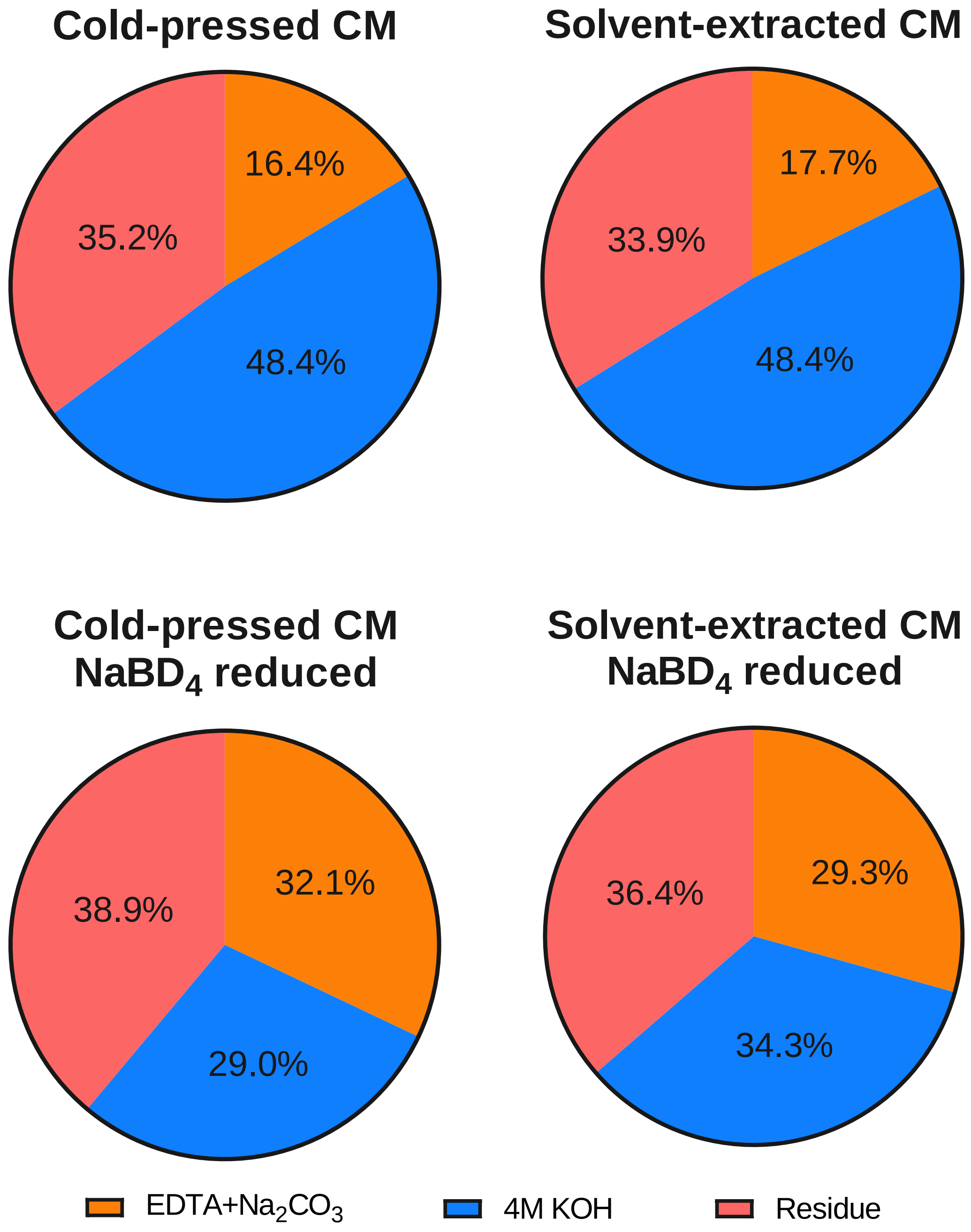
Cold-pressed CM
16.4%
35.2%
48.4%
Solvent-extracted CM
17.7%
33.9%
48.4%
Cold-pressed CM
NaBD4 reduced
32.1%
38.9%
29.0%
Solvent-extracted CM
NaBD4 reduced
29.3%
36.4%
34.3%
EDTA+Na2CO3
4M KOH
Residue
