# Supplementary material for: Combinatorial Glycomic Analyses to Direct CAZyme Discovery for the Tailored Degradation of Canola Meal Non-Starch Dietary Polysaccharides
Source: Microorganisms. 2020 Nov 29;8(12):1888. doi: 10.3390/microorganisms8121888 (PMC7761036; doi:10.3390/microorganisms8121888)
Supplement: Supplementary file 1 [file microorganisms-08-01888-s001.zip › Supplemental Figures.docx]

Supplemental Figures

**
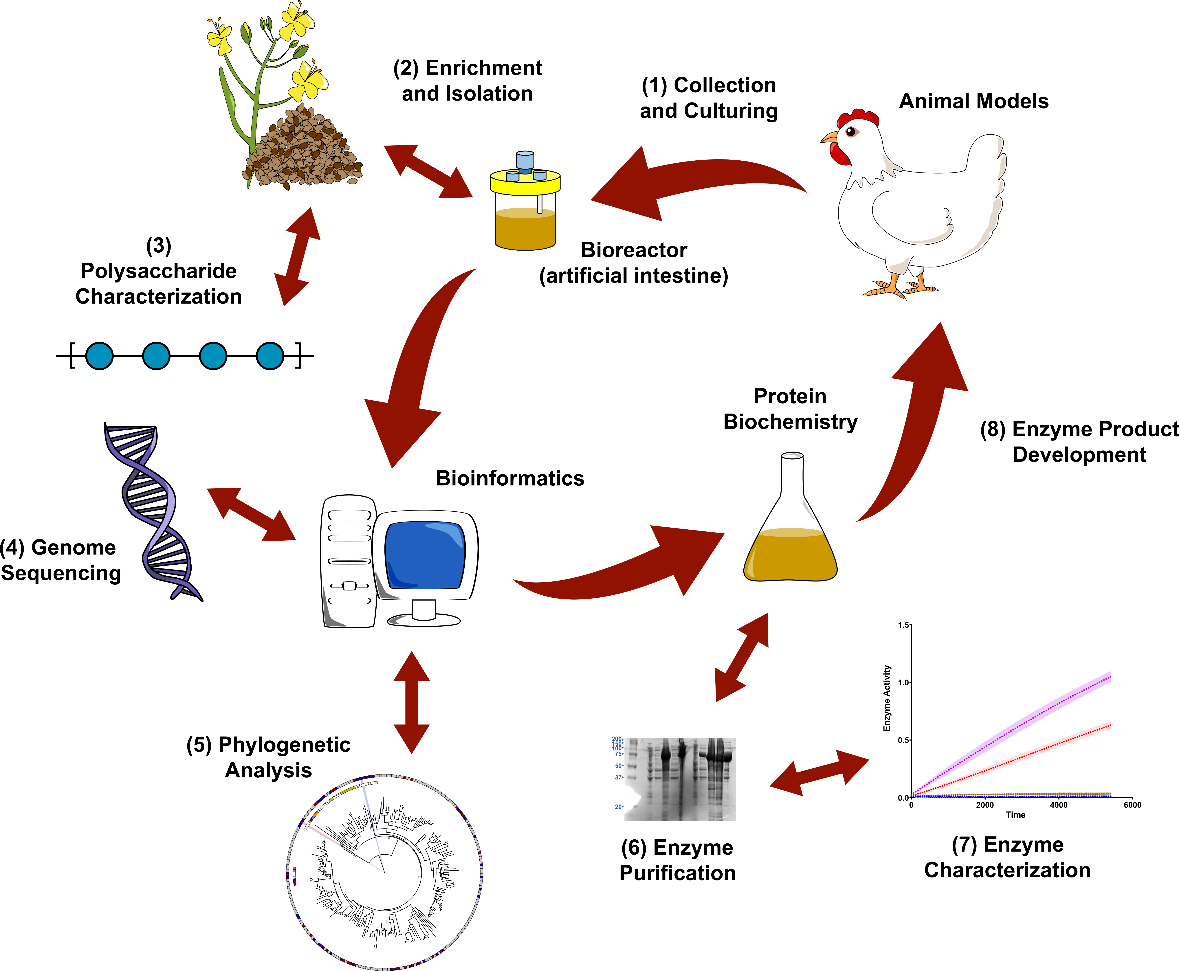
**

**Supplemental Figure S1.** Enzyme discovery platform for the tailored degradation of non-starch dietary polysaccharides. (**1**) Microorganisms are collected from chickens raised on feed-enriched diets. (**2**) Cultured microorganisms are grown on alternative feed stocks to enrich for microbial species which specialize in digesting such feed. (**3**) Glycomics analyses characterizes the polysaccharides within the dietary fibre. (**4**) Isolated microorganisms are sequenced to identify the organism and its carbohydrate-active enzyme (CAZyme) profile. (**5**) SACCHARIS is used to generate enzyme family trees to help identify function of CAZymes. (**6**) Enzymes are recombinantly expressed in *E. coli* and purified, optimizing protocols for enzyme yield and stability. The enzyme structure may determine to identify how the enzyme works to break down complex nutrients. (**7**) Assays for screening enzyme function and determining the kinetics of promising targets are performed. Attempts are made towards improving the rate of degradation. (**8**) Production of promising enzymes are scaled-up and evaluated in animal models.

**
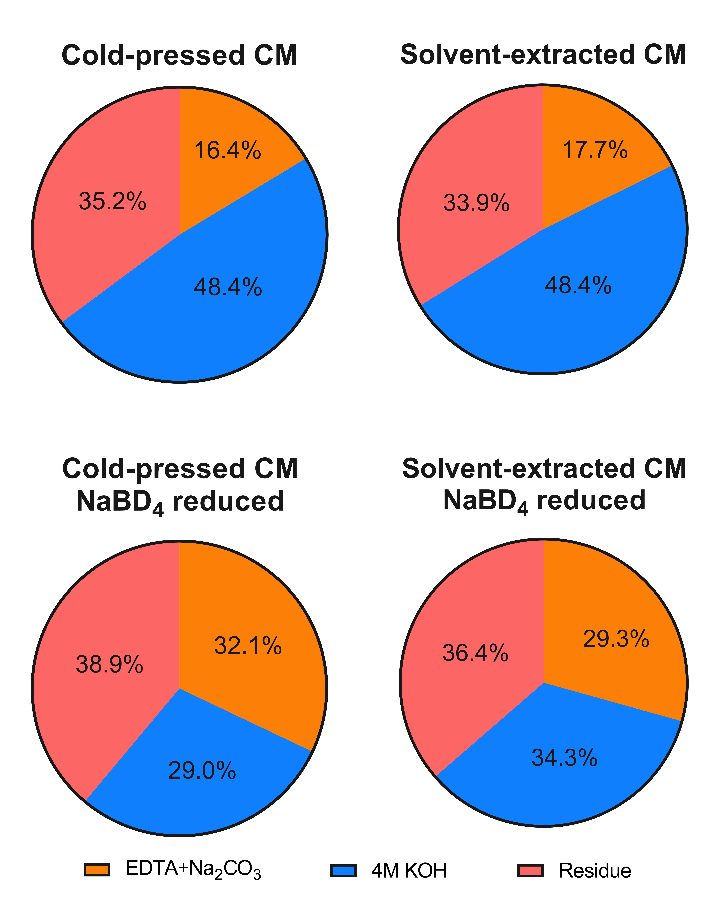
**

**Supplemental Figure S2.** Fractionated yield of cold-pressed and solvent-extracted canola meal (CM) plant cell wall extractions for glycomic linkage analyses.

**
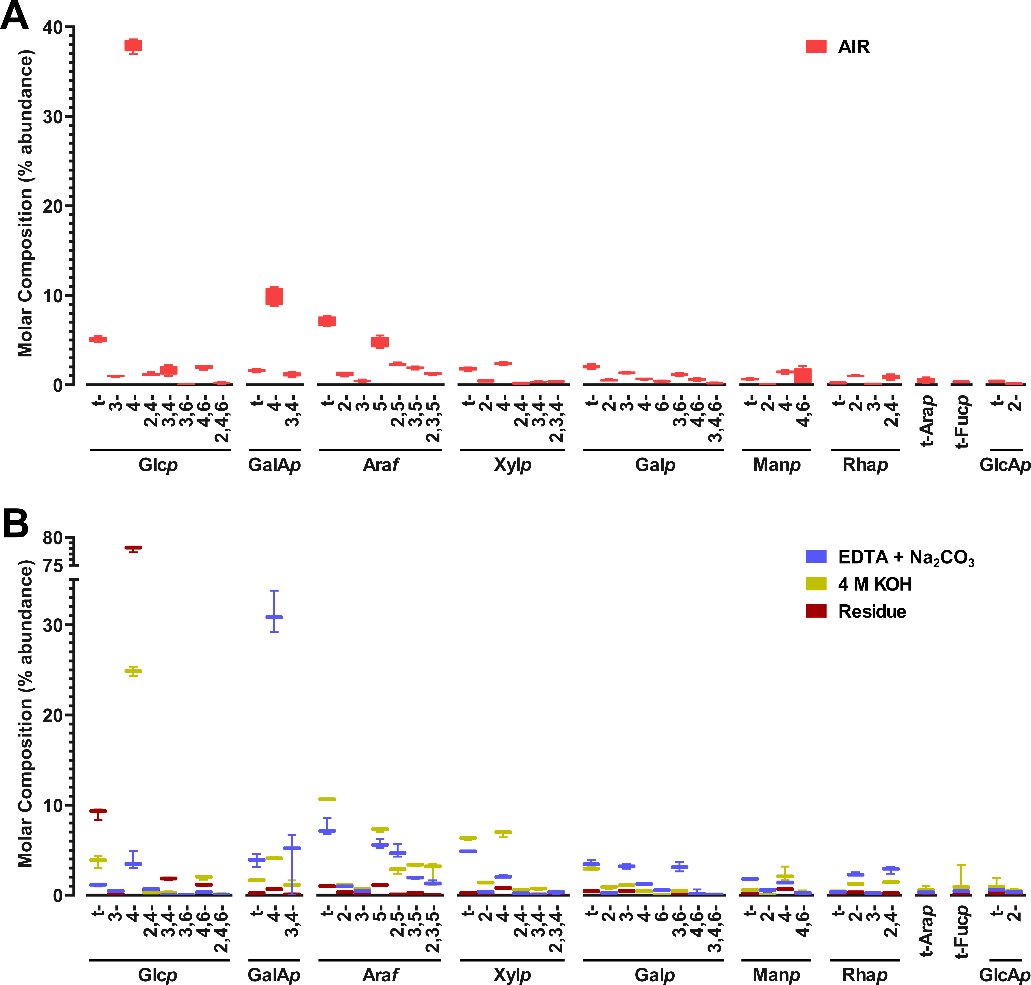
**

**Supplemental Figure S3.** Cell wall linkage composition of solvent-extracted canola meal. Calculated relative composition of linkages (molar %) for solvent-extracted (**A**) CM alcohol insoluble residue (AIR) and (**B**) fractionated extracts of EDTA + Na2CO3, 4 M KOH, or residue. A pre-treatment of NaBD_4_ was conducted to the cell wall before its sequential fractionation in order to increase the extricability of polysaccharides. Four separate experiments were conducted to cell wall and three to each fraction. Values are averages from the separate experiments, presented as bar and whiskers with error bars representing the standard deviation from the mean.

**
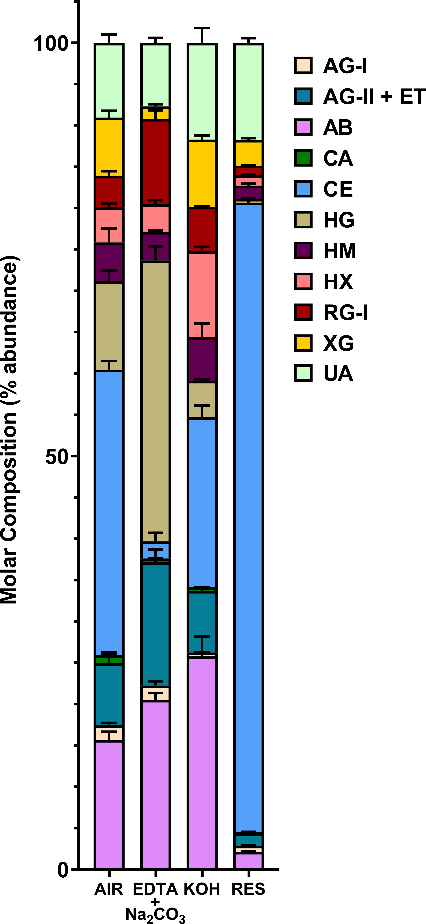
**

**Supplemental Figure S4.** Estimated polysaccharide composition of solvent-extracted canola meal. The composition of polysaccharides was estimated based on linkage composition data (Supplemental Figure S2 and Supplemental Table S2) by assigning the linkages of uronic acids and neutral sugars to polysaccharides (Table 1), according to published procedure [36]. A pre-treatment of NaBD_4_ was conducted to the cell wall before its sequential fractionation in order to increase the extricability of polysaccharides. Four separate experiments conducted to cell wall and three to each fraction. Box and whiskers plots are shown overlaid, with a mean value from the separate experiments represented as a horizontal bar and bars depicting the standard deviation from the mean. The polysaccharides were characterized from compositional linkages as in Table 1: arabinan (AB), type I arabinogalactan (AG-I), type II arabinogalactan (AG-II) and extensin (ET), heteroxylan (HX), xyloglucan (XG), callose (CA), cellulose (CE), rhamnogalacturonan I (RG-I), homogalacturonan (HG), heteromannan (HM), and unassigned linkages (UA).

**
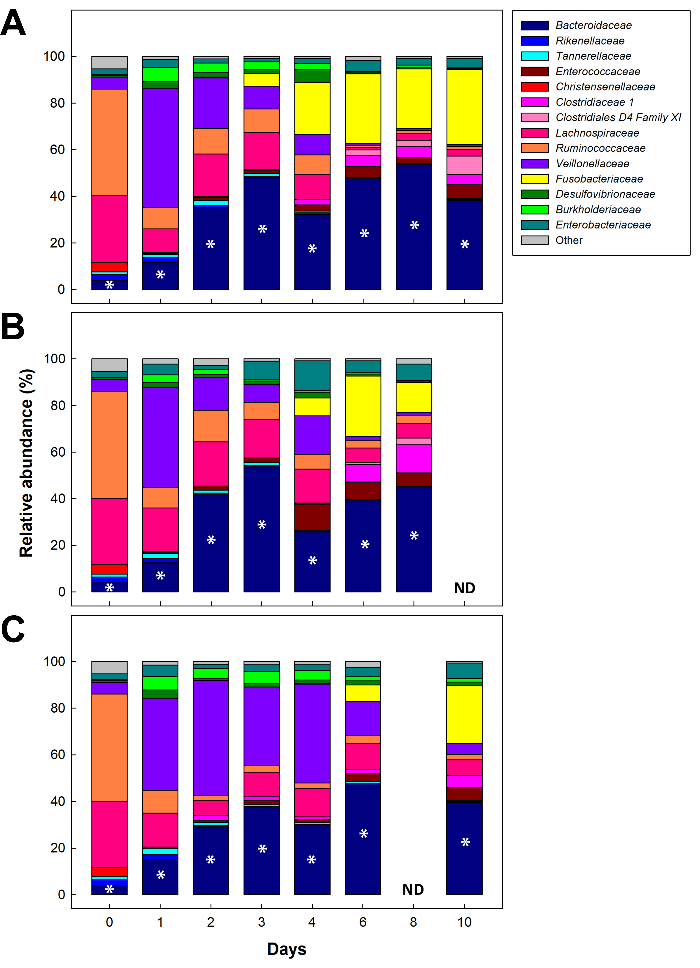
**

**Supplemental Figure S5.** Temporal relative abundance (%) of primary bacterial families in control (CON), NSP, and CM treatment bio-reactor vessels determined by Illumina sequence analysis. ND is not determined. Replicate vessels were combined for Illumina characterization of bacterial communities, and data for day 0 (i.e. before addition of CM and NSP) were averaged. Asterisks show the abundance of bacterial taxa within the family *Bacteroidaceae* (primarily *Bacteroides* spp.).
