# Supplementary material for: Combinatorial Glycomic Analyses to Direct CAZyme Discovery for the Tailored Degradation of Canola Meal Non-Starch Dietary Polysaccharides
Source: Microorganisms. 2020 Nov 29;8(12):1888. doi: 10.3390/microorganisms8121888 (PMC7761036; doi:10.3390/microorganisms8121888)
Supplement: Supplementary file 1 [file microorganisms-08-01888-s001.zip › Supplemental Tables.docx]

Supplemental Tables

**Supplemental Table S1.** Linkage composition of cold-pressed canola meal whole cell wall (AIR) and fractions

|  | **AIR** | | | |  | **EDTA** | | |  | **4 M KOH** | | |  | **Residue** | | |
| --- | --- | --- | --- | --- | --- | --- | --- | --- | --- | --- | --- | --- | --- | --- | --- | --- |
| **t-Ara*f*** | 7.31 | 6.94 | 8.17 | 5.91 |  | 10.74 | 8.58 | 8.80 |  | 14.76 | 14.26 | 13.35 |  | 1.07 | 1.05 | 0.89 |
| **2-Ara*f*** | 0.94 | 0.78 | 1.05 | 0.93 |  | 1.10 | 1.07 | 1.31 |  | 0.81 | 0.81 | 1.08 |  | 0.34 | 0.26 | 0.21 |
| **3-Ara*f*** | 0.42 | 0.33 | 0.39 | 0.42 |  | 0.50 | 0.66 | 0.56 |  | 0.75 | 0.78 | 1.07 |  | 0.21 | 0.22 | 0.20 |
| **5-Ara*f*** | 4.82 | 4.89 | 5.31 | 4.96 |  | 6.84 | 7.29 | 7.86 |  | 10.35 | 9.95 | 8.44 |  | 1.10 | 1.19 | 1.10 |
| **2,5-Ara*f*** | 1.83 | 1.56 | 1.84 | 1.84 |  | 4.51 | 5.14 | 6.26 |  | 3.65 | 3.73 | 2.28 |  | 0.14 | 0.12 | 0.10 |
| **3,5-Ara*f*** | 2.76 | 2.68 | 2.92 | 3.03 |  | 4.22 | 4.45 | 4.53 |  | 7.74 | 7.55 | 5.53 |  | 0.35 | 0.30 | 0.38 |
| **2,3,5-Ara*f*** | 1.73 | 1.69 | 2.09 | 2.03 |  | 2.34 | 2.77 | 3.43 |  | 3.71 | 3.85 | 2.40 |  | 0.05 | 0.07 | 0.05 |
| **t-Ara*p*** | 0.19 | 0.16 | 2.17 | 0.27 |  | 0.31 | 0.34 | 0.36 |  | 0.75 | 0.39 | 0.69 |  | 0.07 | 0.10 | 0.06 |
| **t-Fuc*p*** | 0.43 | 0.43 | 0.45 | 0.46 |  | 0.36 | 0.31 | 0.25 |  | 1.04 | 1.04 | 1.20 |  | 0.10 | 0.08 | 0.08 |
| **t-Gal*p*** | 2.01 | 1.98 | 1.94 | 1.84 |  | 2.53 | 2.80 | 2.50 |  | 3.03 | 3.06 | 3.08 |  | 0.59 | 0.62 | 0.65 |
| **2-Gal*p*** | 0.69 | 0.68 | 0.63 | 0.71 |  | 0.25 | 0.30 | 0.15 |  | 1.47 | 1.42 | 0.80 |  | 0.09 | 0.08 | 0.06 |
| **3-Gal*p*** | 0.88 | 0.79 | 0.88 | 0.92 |  | 2.47 | 2.73 | 2.41 |  | 1.17 | 1.16 | 0.86 |  | 0.39 | 0.36 | 0.39 |
| **4-Gal*p*** | 0.74 | 0.74 | 0.64 | 0.75 |  | 1.36 | 1.39 | 1.32 |  | 0.18 | 0.25 | 0.21 |  | 0.41 | 0.44 | 0.45 |
| **6-Gal*p*** | 0.19 | 0.17 | 0.21 | 0.21 |  | 0.11 | 0.16 | 0.25 |  | 0.10 | 0.08 | 0.27 |  | 0.00 | 0.00 | 0.00 |
| **3,6-Gal*p*** | 0.61 | 0.57 | 0.61 | 0.63 |  | 2.85 | 2.57 | 3.10 |  | 0.04 | 0.05 | 0.20 |  | 0.25 | 0.22 | 0.23 |
| **4,6-Gal*p*** | 0.51 | 0.55 | 0.47 | 0.84 |  | 0.24 | 0.12 | 0.40 |  | 0.00 | 0.00 | 0.00 |  | 0.18 | 0.11 | 0.15 |
| **3,4,6-Gal*p*** | 0.17 | 0.17 | 0.20 | 0.23 |  | 0.14 | 0.09 | 0.30 |  | 0.04 | 0.03 | 0.02 |  | 0.00 | 0.00 | 0.00 |
| **t-GalA*p*** | 1.30 | 1.41 | 1.34 | 1.19 |  | 3.27 | 2.42 | 3.44 |  | 1.72 | 1.66 | 2.37 |  | 0.25 | 0.25 | 0.26 |
| **4-GalA*p*** | 12.34 | 12.58 | 10.52 | 11.86 |  | 25.61 | 25.75 | 24.17 |  | 5.84 | 5.90 | 5.94 |  | 0.79 | 0.88 | 0.90 |
| **3,4-GalA*p*** | 1.76 | 1.67 | 1.32 | 1.63 |  | 3.00 | 3.46 | 4.21 |  | 2.18 | 2.37 | 2.40 |  | 0.01 | 0.20 | 0.22 |
| **t-Glc*p*** | 4.06 | 4.29 | 4.30 | 3.61 |  | 2.05 | 2.07 | 1.57 |  | 2.25 | 2.06 | 3.53 |  | 9.01 | 9.71 | 9.69 |
| **3-Glc*p*** | 2.33 | 2.15 | 2.35 | 2.29 |  | 2.92 | 2.82 | 2.63 |  | 3.67 | 3.51 | 3.38 |  | 0.96 | 0.84 | 0.87 |
| **4-Glc*p*** | 36.47 | 37.36 | 34.75 | 35.98 |  | 4.28 | 4.61 | 2.97 |  | 7.07 | 6.96 | 6.39 |  | 76.64 | 75.91 | 76.38 |
| **2,4-Glc*p*** | 0.62 | 0.68 | 0.55 | 0.98 |  | 0.55 | 0.39 | 0.70 |  | 0.38 | 0.38 | 0.49 |  | 0.64 | 0.57 | 0.57 |
| **3,4-Glc*p*** | 0.86 | 0.85 | 0.75 | 1.07 |  | 0.00 | 0.00 | 0.00 |  | 0.18 | 0.17 | 0.26 |  | 1.74 | 1.82 | 1.93 |
| **3,6-Glc*p*** | 0.15 | 0.14 | 0.15 | 0.18 |  | 0.09 | 0.41 | 0.14 |  | 0.19 | 0.23 | 0.15 |  | 0.07 | 0.11 | 0.05 |
| **4,6-Glc*p*** | 2.72 | 3.06 | 2.53 | 3.20 |  | 0.44 | 0.40 | 0.35 |  | 3.74 | 3.85 | 2.60 |  | 1.23 | 1.27 | 1.29 |
| **2,4,6-Glc*p*** | 0.20 | 0.19 | 0.19 | 0.21 |  | 0.00 | 0.00 | 0.00 |  | 0.09 | 0.08 | 0.12 |  | 0.03 | 0.03 | 0.02 |
| **t-GlcA*p*** | 0.35 | 0.38 | 0.32 | 0.32 |  | 0.55 | 0.64 | 0.93 |  | 0.69 | 0.62 | 0.90 |  | 0.23 | 0.24 | 0.25 |
| **2-GlcA*p*** | 0.11 | 0.08 | 0.11 | 0.14 |  | 0.28 | 0.37 | 0.23 |  | 0.37 | 0.38 | 0.72 |  | 0.11 | 0.09 | 0.07 |
| **t-Man*p*** | 0.48 | 0.54 | 0.52 | 0.50 |  | 1.47 | 1.53 | 1.28 |  | 0.69 | 0.68 | 1.15 |  | 0.19 | 0.24 | 0.25 |
| **2-Man*p*** | 0.00 | 0.00 | 0.00 | 0.00 |  | 0.72 | 0.62 | 0.96 |  | 0.00 | 0.00 | 0.00 |  | 0.00 | 0.00 | 0.00 |
| **4-Man*p*** | 1.33 | 1.24 | 1.24 | 1.43 |  | 2.21 | 2.36 | 2.41 |  | 1.47 | 1.47 | 1.18 |  | 0.79 | 0.69 | 0.67 |
| **4,6-Man*p*** | 0.36 | 0.38 | 0.30 | 0.41 |  | 0.25 | 0.34 | 0.29 |  | 0.43 | 0.49 | 0.27 |  | 0.12 | 0.10 | 0.11 |
| **t-Rha*p*** | 0.25 | 0.21 | 0.21 | 0.18 |  | 0.34 | 0.18 | 0.34 |  | 0.42 | 0.43 | 0.87 |  | 0.07 | 0.07 | 0.06 |
| **2-Rha*p*** | 0.88 | 0.78 | 0.79 | 0.78 |  | 2.15 | 2.26 | 1.97 |  | 1.22 | 1.28 | 1.32 |  | 0.32 | 0.30 | 0.29 |
| **3-Rha*p*** | 0.10 | 0.11 | 0.07 | 0.08 |  | 0.18 | 0.14 | 0.16 |  | 0.20 | 0.21 | 0.30 |  | 0.00 | 0.00 | 0.00 |
| **2,4-Rha*p*** | 0.80 | 1.15 | 0.85 | 1.08 |  | 1.92 | 2.51 | 1.47 |  | 1.09 | 1.18 | 1.42 |  | 0.11 | 0.18 | 0.16 |
| **t-Xyl*p*** | 2.51 | 1.85 | 2.41 | 2.06 |  | 3.35 | 2.70 | 2.86 |  | 7.30 | 7.94 | 11.18 |  | 0.29 | 0.25 | 0.14 |
| **2-Xyl*p*** | 0.52 | 0.40 | 0.56 | 0.56 |  | 0.34 | 0.31 | 0.36 |  | 1.68 | 1.91 | 1.97 |  | 0.10 | 0.09 | 0.06 |
| **4-Xyl*p*** | 2.05 | 1.77 | 2.01 | 1.97 |  | 2.36 | 2.26 | 2.11 |  | 6.69 | 6.77 | 8.30 |  | 0.83 | 0.83 | 0.61 |
| **2,4-Xyl*p*** | 0.15 | 0.14 | 0.16 | 0.23 |  | 0.20 | 0.21 | 0.22 |  | 0.45 | 0.51 | 0.48 |  | 0.04 | 0.03 | 0.02 |
| **3,4-Xyl*p*** | 0.12 | 0.15 | 0.13 | 0.15 |  | 0.07 | 0.07 | 0.10 |  | 0.25 | 0.29 | 0.56 |  | 0.06 | 0.05 | 0.07 |
| **2,3,4-Xyl*p*** | 0.91 | 1.34 | 1.59 | 1.93 |  | 0.53 | 0.37 | 0.32 |  | 0.15 | 0.23 | 0.26 |  | 0.03 | 0.02 | 0.04 |

Note: Cell wall was treated with NaBD_4_ prior to the sequential fractionation. Four separate experiments were conducted to cell wall, and three to each fraction.

**Supplemental Table S2.** Linkage composition of solvent-extracted canola meal whole cell wall (AIR) and fractions

|  | **AIR** | | | |  | **EDTA** | | |  | **4 M KOH** | | |  | **Residue** | | |
| --- | --- | --- | --- | --- | --- | --- | --- | --- | --- | --- | --- | --- | --- | --- | --- | --- |
| **t-Ara*f*** | 6.60 | 7.66 | 6.57 | 7.62 |  | 7.13 | 8.60 | 6.80 |  | 10.71 | 10.64 | 10.62 |  | 1.00 | 0.90 | 1.01 |
| **2-Ara*f*** | 1.24 | 1.49 | 0.94 | 1.48 |  | 0.90 | 0.97 | 0.98 |  | 1.15 | 1.13 | 1.17 |  | 0.35 | 0.31 | 0.33 |
| **3-Ara*f*** | 0.41 | 0.61 | 0.31 | 0.47 |  | 0.37 | 0.47 | 0.46 |  | 0.67 | 0.79 | 0.69 |  | 0.22 | 0.13 | 0.27 |
| **5-Ara*f*** | 4.14 | 5.52 | 4.80 | 4.36 |  | 5.59 | 5.24 | 6.25 |  | 7.05 | 7.36 | 7.30 |  | 1.22 | 0.99 | 1.13 |
| **2,5-Ara*f*** | 2.26 | 2.51 | 2.27 | 2.22 |  | 4.29 | 4.67 | 5.65 |  | 2.39 | 3.01 | 2.86 |  | 0.09 | 0.07 | 0.08 |
| **3,5-Ara*f*** | 2.00 | 1.92 | 1.72 | 2.07 |  | 1.80 | 1.99 | 1.95 |  | 3.26 | 3.39 | 3.48 |  | 0.26 | 0.22 | 0.28 |
| **2,3,5-Ara*f*** | 1.34 | 1.40 | 1.07 | 1.37 |  | 1.63 | 1.30 | 1.25 |  | 1.13 | 3.49 | 3.19 |  | 0.06 | 0.08 | 0.07 |
| **t-Ara*p*** | 0.67 | 0.30 | 0.25 | 0.84 |  | 0.19 | 0.42 | 0.31 |  | 1.04 | 0.51 | 0.57 |  | 0.06 | 0.06 | 0.08 |
| **t-Fuc*p*** | 0.37 | 0.31 | 0.35 | 0.39 |  | 0.45 | 0.45 | 0.41 |  | 3.31 | 0.88 | 0.80 |  | 0.12 | 0.12 | 0.10 |
| **t-Gal*p*** | 2.05 | 2.30 | 2.17 | 1.79 |  | 3.42 | 3.33 | 3.87 |  | 3.08 | 2.95 | 2.83 |  | 0.45 | 0.43 | 0.50 |
| **2-Gal*p*** | 0.64 | 0.50 | 0.50 | 0.47 |  | 0.28 | 0.36 | 0.18 |  | 0.69 | 0.96 | 0.92 |  | 0.06 | 0.03 | 0.07 |
| **3-Gal*p*** | 1.49 | 1.40 | 1.26 | 1.36 |  | 3.19 | 2.95 | 3.43 |  | 1.20 | 1.07 | 1.11 |  | 0.49 | 0.47 | 0.48 |
| **4-Gal*p*** | 0.75 | 0.72 | 0.70 | 0.54 |  | 1.24 | 1.12 | 1.32 |  | 0.42 | 0.47 | 0.44 |  | 0.43 | 0.37 | 0.41 |
| **6-Gal*p*** | 0.49 | 0.45 | 0.42 | 0.29 |  | 0.70 | 0.54 | 0.53 |  | 0.05 | 0.02 | 0.09 |  | 0.00 | 0.00 | 0.00 |
| **3,6-Gal*p*** | 1.13 | 1.36 | 1.12 | 1.02 |  | 3.10 | 2.64 | 3.70 |  | 0.41 | 0.49 | 0.46 |  | 0.25 | 0.17 | 0.24 |
| **4,6-Gal*p*** | 0.81 | 0.49 | 0.43 | 0.74 |  | 0.64 | 0.15 | 0.10 |  | 0.00 | 0.00 | 0.00 |  | 0.18 | 0.15 | 0.09 |
| **3,4,6-Gal*p*** | 0.27 | 0.15 | 0.11 | 0.24 |  | 0.05 | 0.08 | 0.06 |  | 0.09 | 0.00 | 0.08 |  | 0.00 | 0.00 | 0.00 |
| **t-GalA*p*** | 1.60 | 1.53 | 1.81 | 1.40 |  | 3.12 | 4.59 | 3.92 |  | 1.78 | 1.66 | 1.58 |  | 0.21 | 0.28 | 0.23 |
| **4-GalA*p*** | 10.91 | 9.11 | 10.48 | 8.86 |  | 30.84 | 29.23 | 33.79 |  | 3.99 | 4.25 | 4.09 |  | 0.67 | 0.65 | 0.71 |
| **3,4-GalA*p*** | 1.47 | 0.89 | 1.20 | 1.08 |  | 6.66 | 5.20 | 0.04 |  | 1.08 | 1.16 | 1.64 |  | 0.09 | 0.09 | 0.18 |
| **t-Glc*p*** | 4.92 | 4.80 | 4.94 | 5.51 |  | 0.98 | 1.17 | 1.28 |  | 4.32 | 3.88 | 3.04 |  | 8.33 | 9.32 | 9.50 |
| **3-Glc*p*** | 1.07 | 0.95 | 0.91 | 1.07 |  | 0.46 | 0.48 | 0.49 |  | 0.47 | 0.42 | 0.43 |  | 0.15 | 0.12 | 0.12 |
| **4-Glc*p*** | 36.98 | 38.60 | 38.27 | 38.14 |  | 3.45 | 4.93 | 3.00 |  | 24.85 | 25.36 | 24.35 |  | 78.33 | 78.17 | 77.30 |
| **2,4-Glc*p*** | 1.21 | 0.99 | 0.96 | 1.41 |  | 0.77 | 0.61 | 0.70 |  | 0.36 | 0.24 | 0.30 |  | 0.63 | 0.55 | 0.54 |
| **3,4-Glc*p*** | 1.56 | 1.02 | 2.20 | 2.05 |  | 0.00 | 0.00 | 0.00 |  | 0.30 | 0.20 | 0.42 |  | 1.86 | 1.96 | 1.65 |
| **3,6-Glc*p*** | 0.00 | 0.00 | 0.00 | 0.00 |  | 0.00 | 0.00 | 0.00 |  | 0.00 | 0.00 | 0.00 |  | 0.00 | 0.00 | 0.00 |
| **4,6-Glc*p*** | 2.27 | 1.92 | 1.70 | 2.22 |  | 0.33 | 0.33 | 0.22 |  | 2.09 | 1.73 | 2.06 |  | 1.19 | 1.16 | 1.02 |
| **2,4,6-Glc*p*** | 0.32 | 0.22 | 0.12 | 0.26 |  | 0.00 | 0.00 | 0.00 |  | 0.00 | 0.20 | 0.08 |  | 0.02 | 0.02 | 0.03 |
| **t-GlcA*p*** | 0.41 | 0.44 | 0.46 | 0.38 |  | 0.62 | 0.61 | 0.62 |  | 0.82 | 0.96 | 1.91 |  | 0.27 | 0.39 | 0.13 |
| **2-GlcA*p*** | 0.20 | 0.09 | 0.11 | 0.09 |  | 0.35 | 0.28 | 0.36 |  | 0.24 | 0.69 | 0.46 |  | 0.07 | 0.05 | 0.07 |
| **t-Man*p*** | 0.82 | 0.56 | 0.51 | 0.64 |  | 1.81 | 1.89 | 1.75 |  | 0.63 | 0.56 | 0.61 |  | 0.18 | 0.20 | 0.22 |
| **2-Man*p*** | 0.00 | 0.00 | 0.00 | 0.00 |  | 0.39 | 0.57 | 0.58 |  | 0.00 | 0.00 | 0.00 |  | 0.00 | 0.00 | 0.00 |
| **4-Man*p*** | 1.64 | 1.23 | 1.45 | 1.53 |  | 1.40 | 1.39 | 1.52 |  | 2.08 | 1.69 | 3.17 |  | 0.68 | 0.61 | 0.70 |
| **4,6-Man*p*** | 0.20 | 1.06 | 2.11 | 0.19 |  | 0.28 | 0.27 | 0.31 |  | 0.53 | 0.17 | 0.30 |  | 0.09 | 0.08 | 0.23 |
| **t-Rha*p*** | 0.23 | 0.19 | 0.25 | 0.26 |  | 0.37 | 0.39 | 0.24 |  | 0.51 | 0.41 | 0.42 |  | 0.06 | 0.06 | 0.07 |
| **2-Rha*p*** | 0.96 | 0.98 | 1.12 | 0.92 |  | 2.29 | 2.21 | 2.53 |  | 1.33 | 1.23 | 1.26 |  | 0.31 | 0.32 | 0.35 |
| **3-Rha*p*** | 0.13 | 0.05 | 0.07 | 0.12 |  | 0.24 | 0.20 | 0.22 |  | 0.20 | 0.21 | 0.20 |  | 0.00 | 0.00 | 0.00 |
| **2,4-Rha*p*** | 0.99 | 1.19 | 0.88 | 0.58 |  | 2.94 | 2.39 | 3.10 |  | 1.32 | 1.43 | 1.45 |  | 0.23 | 0.24 | 0.25 |
| **t-Xyl*p*** | 1.82 | 1.55 | 1.95 | 1.92 |  | 4.86 | 4.89 | 4.81 |  | 6.35 | 6.48 | 6.13 |  | 0.29 | 0.24 | 0.31 |
| **2-Xyl*p*** | 0.41 | 0.44 | 0.46 | 0.48 |  | 0.40 | 0.31 | 0.39 |  | 1.45 | 1.40 | 1.38 |  | 0.10 | 0.08 | 0.11 |
| **4-Xyl*p*** | 2.40 | 2.22 | 2.31 | 2.59 |  | 2.04 | 2.04 | 2.23 |  | 6.99 | 7.03 | 6.42 |  | 0.80 | 0.73 | 0.94 |
| **2,4-Xyl*p*** | 0.15 | 0.19 | 0.16 | 0.18 |  | 0.21 | 0.21 | 0.27 |  | 0.51 | 0.70 | 0.58 |  | 0.04 | 0.04 | 0.04 |
| **3,4-Xyl*p*** | 0.36 | 0.24 | 0.24 | 0.43 |  | 0.07 | 0.09 | 0.07 |  | 0.77 | 0.54 | 0.74 |  | 0.08 | 0.07 | 0.08 |
| **2,3,4-Xyl*p*** | 0.33 | 0.45 | 0.31 | 0.44 |  | 0.13 | 0.45 | 0.32 |  | 0.41 | 0.24 | 0.40 |  | 0.06 | 0.05 | 0.06 |

Note: Cell wall was treated with NaBD_4_ prior to the sequential fractionation. Four separate experiments were conducted to cell wall, and three to each fraction.

**Supplemental Table S3.** CAZome of bacterial isolates for the GH5 and GH43 subfamilies

| **CAZy family** | **Major known activity** | ***B. theta* CMU13** | ***B. theta* CMU108** | ***B. ovatus* CMU19** | ***B. ovatus* CMU33** | ***B. fragilis* CMU36** | ***B. fragilis* CMU103** |
| --- | --- | --- | --- | --- | --- | --- | --- |
|  |  |  |  |  |  |  |  |
| GH5 | cellulase | 1 | 1 | 0 | 0 | 0 | 0 |
| GH5_13 |  | 0 | 0 | 3 | 3 | 0 | 0 |
| GH5_5 |  | 0 | 0 | 2 | 2 | 0 | 0 |
| GH5_2 |  | 0 | 0 | 1 | 1 | 0 | 0 |
| GH5_4 |  | 0 | 0 | 1 | 1 | 0 | 0 |
| GH5_7 |  | 0 | 0 | 0 | 0 | 1 | 1 |
|  | **Total** | **1** | **1** | **7** | **7** | **1** | **1** |
|  |  |  |  |  |  |  |  |
| GH43 | arabinases & xylosidases | 4 | 4 | 5 | 5 | 3 | 3 |
| GH43_24 |  | 6 | 6 | 6 | 6 | 1 | 1 |
| GH43_10 |  | 4 | 4 | 3 | 3 | 1 | 1 |
| GH43_31 |  | 3 | 3 | 1 | 1 | 1 | 1 |
| GH43_2 |  | 2 | 2 | 4 | 4 | 0 | 0 |
| GH43_18 |  | 2 | 2 | 3 | 3 | 0 | 0 |
| GH43_3 |  | 2 | 2 | 3 | 3 | 1 | 1 |
| GH43_26 |  | 2 | 2 | 2 | 2 | 2 | 2 |
| GH43_19 |  | 2 | 2 | 1 | 1 | 0 | 0 |
| GH43_4 |  | 2 | 2 | 0 | 0 | 0 | 0 |
| GH43_29 |  | 1 | 1 | 2 | 2 | 0 | 0 |
| GH43_17 |  | 1 | 1 | 1 | 1 | 0 | 0 |
| GH43_8 |  | 1 | 1 | 1 | 1 | 0 | 0 |
| GH43_9 |  | 1 | 1 | 0 | 0 | 0 | 0 |
| GH43_12 |  | 0 | 0 | 4 | 4 | 0 | 0 |
| GH43_28 |  | 0 | 0 | 2 | 2 | 1 | 1 |
| GH43_1 |  | 0 | 0 | 1 | 1 | 0 | 0 |
|  | **Total** | **33** | **33** | **39** | **39** | **10** | **10** |
