# Supplementary figures and images for: Combinatorial Glycomic Analyses to Direct CAZyme Discovery for the Tailored Degradation of Canola Meal Non-Starch Dietary Polysaccharides
Source: Microorganisms. 2020 Nov 29;8(12):1888. doi: 10.3390/microorganisms8121888 (PMC7761036; doi:10.3390/microorganisms8121888)

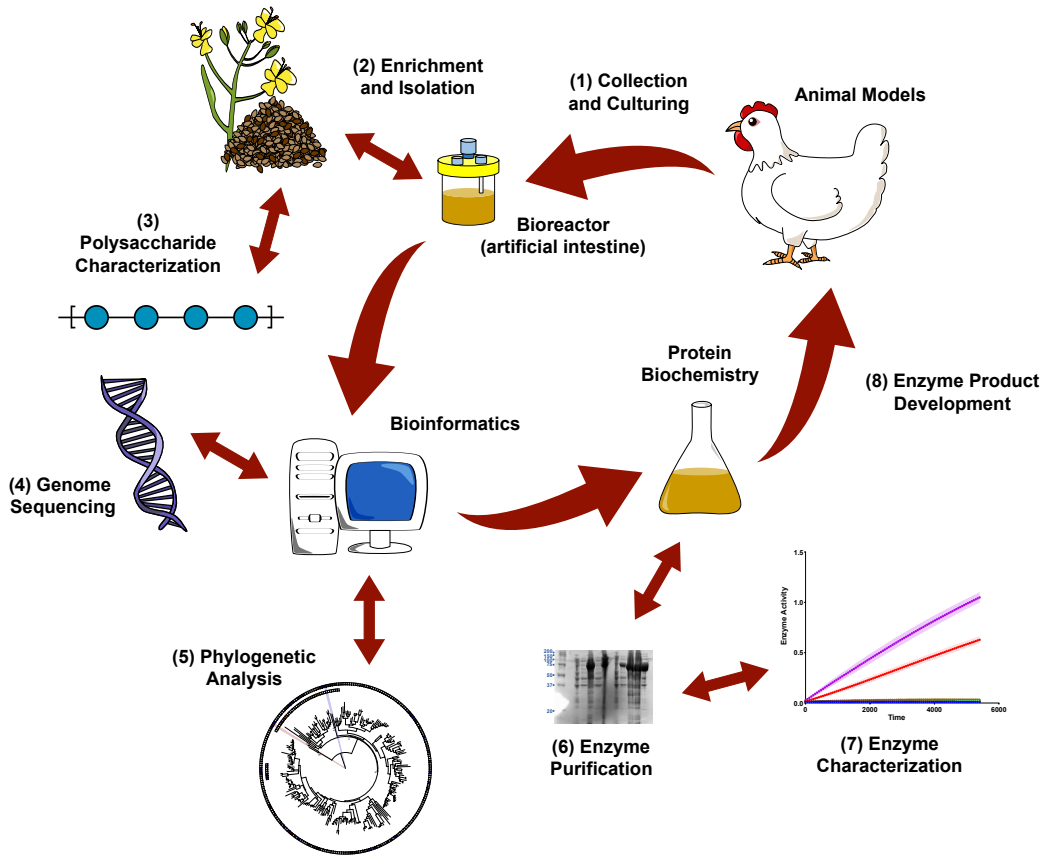

Supplement: Supplementary file 1 [file microorganisms-08-01888-s001.zip › FigS1_pipeline.pdf]

**A**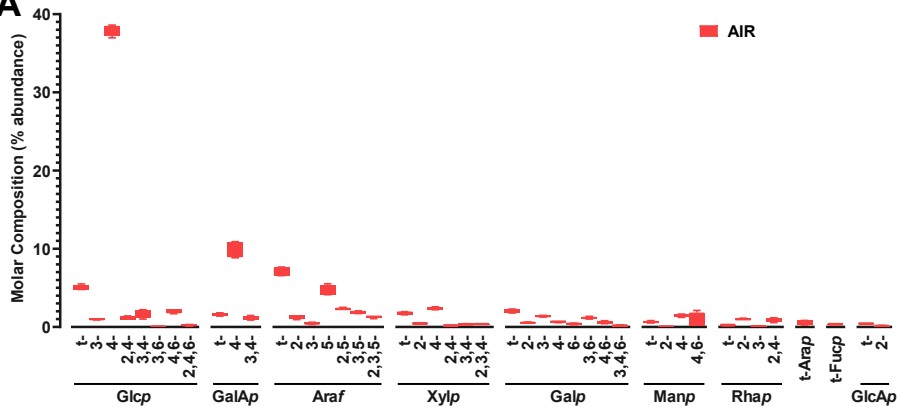**B**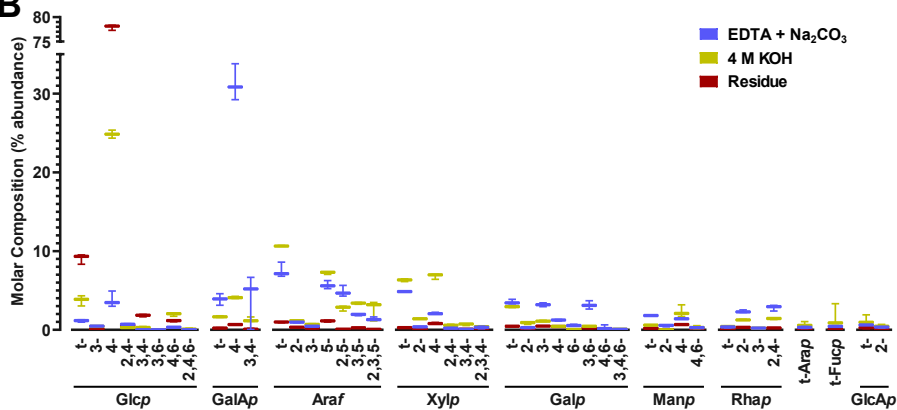

Supplement: Supplementary file 1 [file microorganisms-08-01888-s001.zip › FigS3_linkages_SE.pdf]

Molar Composition (% abundance)

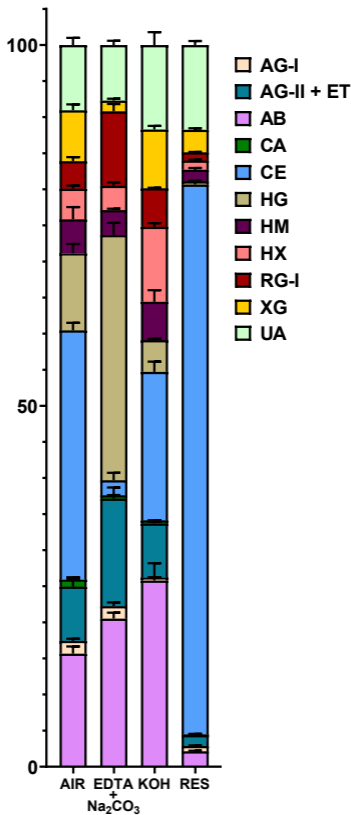

Supplement: Supplementary file 1 [file microorganisms-08-01888-s001.zip › FigS4_poly_comp_SE.pdf]

**A**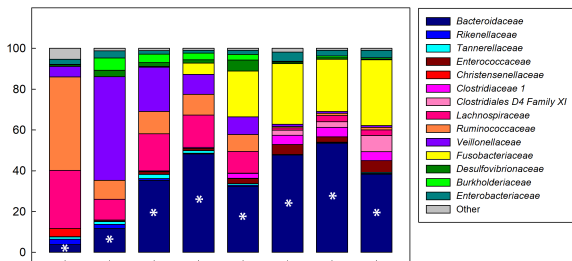**B**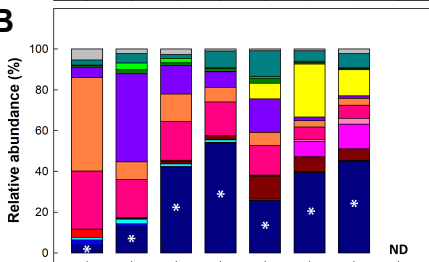**C**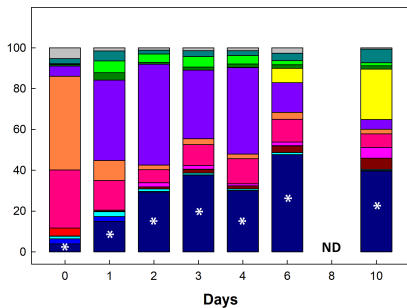

Supplement: Supplementary file 1 [file microorganisms-08-01888-s001.zip › FigS5_family_abundance.pdf]
